# Does observing reciprocity or exploitation affect elevation, a mechanism driving prosociality?

**DOI:** 10.1017/ehs.2019.3

**Published:** 2019-05-23

**Authors:** Daniel M.T. Fessler, Adam Maxwell Sparks, Theodore Samore, Colin Holbrook

**Affiliations:** 1 Department of Anthropology and Center for Behavior, Evolution and Culture, University of California, Los Angeles, Los Angeles, California, USA; 2 Department of Cognitive and Information Sciences, University of California, Merced, Merced, California, USA

**Keywords:** Prosociality, emotion, elevation, exploitation

## Abstract

Fitness is enhanced by determining when to behave prosocially. *Elevation*, an uplifting emotion elicited by witnessing exemplary prosociality, upregulates prosociality in the presence of prosocial others, as such contexts render prosociality profitable and/or antisociality costly. Prior research examines responses to a single highly prosocial individual. However, the profitability of enhancing prosociality hinges not only on potential interactions with a single actor, but also on the actions of others. Accordingly, information regarding how others respond to the prosocial exemplar may influence elevation elicitation and corresponding changes in prosocial motivation. If others reciprocate the exemplar's prosociality, or pay prosociality forward, this expands opportunities for the observer to profit by increasing prosociality, and thus could enhance elevation elicitation. Conversely, if others exploit the exemplar, this may diminish the profitability of prosociality, as the observer who acts prosocially may similarly be exploited and/or the resources with which the exemplar could reciprocate will be depleted. Conducting three online studies of Americans in which information regarding the responses of others to a prosocial exemplar was manipulated, we find that, against predictions, prosocial responses by the beneficiaries of prosociality generally do not enhance elevation among observers, whereas, consonant with predictions, antisocial responses markedly diminish elevation among observers.

## Graphical abstract

**Figure:**
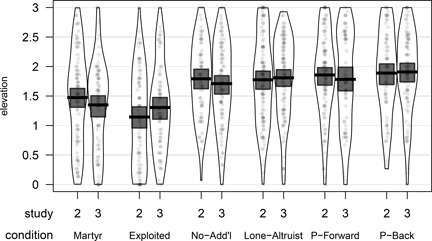

**Media summary:** Elevation, an uplifting response to prosociality, is not enhanced by observing reciprocity, but is diminished by observing exploitation.

## Introduction

A growing literature explores the emotion that Haidt and colleagues (Haidt 2000, 2003a, b; Keltner and Haidt 2003; Algoe and Haidt 2009) termed *elevation*, a positive, uplifting feeling, elicited by witnessing exemplary prosocial behavior, that motivates increased prosociality in the observer (reviewed in Thomson and Siegel 2017; Pohling and Diessner 2016). We theorize that elevation is part of an evolved mechanism that adjusts the actor's inclination to behave prosocially in response to indications that, by virtue of the presence of other prosocial actors, the immediate environment is one in which prosociality will yield benefits. Here, we explore the responses of this mechanism to the social dynamics observed by the actor, asking how the reactions of recipients of prosociality influence elevation elicitation and subsequent prosocial motivation. Specifically, we examine whether the evocative power of the prosocial actions of a single exemplary individual is enhanced by prosocial reactions (in the form of direct or indirect reciprocity) from the beneficiaries of such actions, and conversely, whether antisocial responses to the prosocial actions of an exemplary individual degrade the potential of said actions to elicit elevation. The answers to these questions can shed light on the workings of the elevation mechanism, and have translational implications regarding the potential of different social events to initiate or impede cascades of contagious prosocial behavior.

To summarize our model of elevation, we argue as follows: first, the payoffs to the individual resulting from incurring costs to provide benefits to others (or, less directly, contributing to a larger cooperative enterprise) hinge on the likelihood that such costs will subsequently be outweighed by benefits the actor receives from others in virtue of her actions. To sketch the possibilities in their starkest terms, when, in their immediate surroundings, individuals are amidst people most of whom cooperate for the public good and/or punish free-riders, prosocial acts are more likely to be directly or indirectly reciprocated, and cooperative ventures will generally yield greater gains, as most or all of the relevant parties will invest in the venture. Likewise, behaving in a self-interested, antisocial manner in the presence of prosocial actors will frequently be costly, both because they will exclude one from rewarding cooperative ventures, and because prosocial punishers may punish one for behaving selfishly. Conversely, when the individual is surrounded by a substantial proportion of antisocial others, prosocial acts will rarely be directly or indirectly reciprocated, and instead, the prosocial actor will often be exploited by others. Similarly, behaving in a self-interested, antisocial manner will generally not be punished, as others will not be willing to pay the costs of punishment to enhance security for third parties. Second, holding aside for now the complexities of social dynamics – the topic of the present paper – observing an exemplary prosocial individual is a powerful cue that the current setting is one in which prosociality may be rewarded and antisociality may be punished. Engaging an emotional driver of prosociality in the presence of such an individual thus temporarily upregulates prosocial inclinations in a manner benefiting the actor. Lastly, because any given setting entails some uncertainty regarding how others will respond to prosocial or antisocial actions, interpretations of immediate events are fundamentally colored by previous experience. We argue that actors approach a given event with a representation, based on past experience, of the prior probability that others will behave prosocially. Elsewhere, we extensively demonstrate that this attitude, which we term *idealism*, predicts the extent to which a specific prosocial event elicits elevation.

From its inception, much research on elevation has focused on observers’ reactions to a single exemplary prosocial target (see Thomson and Siegel 2017; Pohling and Diessner 2016). There are several ways that the actions of one person might index the likely profitability of prosocial action, as follows:

First, the prosocial individual might herself constitute a prospective cooperative partner. Elevation may function completely or partially to establish a dyadic partnership with the exemplar. Consistent with this, observers experiencing elevation are positively inclined toward the observed individual, and are motivated to approach and offer praise and other rewards (Algoe and Haidt 2009). However, there is also considerable evidence that prosocial motivations upregulated by elevation are not uniquely focused on the exemplary individual. Indeed, a common finding is that, in both their stated desires and their measured behaviors, elevated participants evince enhanced prosociality toward people unconnected to the observed prosocial actor. (Although stated desires indicate an increase in broadly prosocial motivation, it remains unknown whether elevation-driven prosociality would be directed at third parties if participants could focus their efforts exclusively on the exemplary individual.)

Second, even if the elevation mechanism is not designed to exclusively target the exemplary individual as a cooperative partner, a single observed prosocial individual can nonetheless adaptively upregulate prosociality if their presence is a cue of an environment suitable for other cooperative ventures. In general, individuals who behave in a highly prosocial manner when surrounded by selfish individuals will not persist for long, as costly exploitation without offsetting benefits will force them to desist, leave or be weakened to the point of being unable to continue acting prosocially. Accordingly, the observer can conclude that the presence of an exemplary prosocial individual will frequently index a social environment in which prosociality pays off, and this is especially true if the exemplary individual is seen to persist in prosocial behavior over time.

Third, if witnessing an exemplary prosocial actor increases one's own prosocial actions, these in turn feed into the social milieu. Although much depends on the timecourse of interactions, the number and density of social connections, and the baseline prior attitudes that the interactants bring to the situation, under the right conditions, virtuous cycles of positive feedback can occur whereby an exemplary prosocial actor can push a group toward an equilibrium in which high levels of prosociality are common – a context in which it pays for the observer to join in this virtuous cycle and behave prosocially.

Note that both the second and third possibilities listed above rely on individuals other than the exemplary prosocial actor as the source of benefits making it profitable for the observer to upregulate prosocial motivations. If, per the second possibility, the exemplary prosocial individual indexes a highly prosocial environment, then the observer who acts prosocially will be rewarded not merely by the focal individual, but by others as well; likewise, others are likely to punish selfish behavior by the observer. If, per the third possibility, the presence of the exemplary prosocial individual is informative because of the possibility of a virtuous cycle of increasing prosociality, then the observer who acts prosocially will both contribute to, and benefit from, this cycle, while the observer who acts selfishly may be increasingly punished by others. Of these two circumstances, the third is potentially more expansive in scope than the second, since contagious prosociality can progressively increase the number of prosocial individuals with whom profitable interactions might occur. Lastly, note that, when the number of prosocial actors is sufficiently large and/or their interactions are sufficiently stable over time, a prosocial milieu can be sustained through indirect rather than direct reciprocity, that is, prosocial actors receive benefits not from those whom they benefit, but from third parties who witness or learn of their actions (Alexander 1987; Nowak and Sigmund 2005).

If, per the second and third possibilities, the benefits of upregulating prosocial motivation in the presence of an exemplary prosocial individual stem at least in part from others’ responses to the observer's enhanced prosociality, then information regarding reactions by the focal individual's beneficiaries is relevant, and thus should influence elevation elicitation. Specifically, elevation should be enhanced relative to that elicited by the actions of the exemplary individual if recipients of her prosociality either reciprocate (i.e. pay the prosociality back) or themselves demonstrate high levels of prosociality (i.e. either behave from baseline in an exemplary manner, or else enhance their prosociality, that is, pay the prosociality forward). Of these two circumstances, the latter can be expected to be even more evocative than the former, as a large or ever-expanding set of prosocial individuals enhances the likelihood that the observer who increases his own prosociality will benefit thereby, since payoffs do not depend exclusively on the propensity or capacity of any given recipient to reciprocate.

Via direct or indirect reciprocity, people like those observed responding to the exemplar are one avenue via which elevation-motivated prosociality can pay off. If so, what if, rather than responding to the exemplar by either paying back his generosity or paying it forward, others do neither? This nonresponsiveness may degrade the evocative power of a single prosocial exemplar, as observing such reactions should indicate that payoffs from enhancing one's prosociality may be limited to the focal individual's direct or indirect reciprocity – a narrower source of benefits than the larger community of actors.

Finally, and critically, witnessing others actively exploiting an exemplary prosocial actor should markedly erode the capacity of the latter's actions to evoke elevation and corresponding prosocial motivation in the observer. First, if a prosocial actor is exploited, this will often diminish said actor's ability to reciprocate should the observer act prosocially. Second, observing such exploitation should indicate that not only would any prosocial actions by the observer be less likely to elicit direct or indirect reciprocity from others in the immediate environment, but, moreover, the observer would be more likely to suffer exploitation herself. The presence of selfish, exploitative individuals can lead to a cascade wherein prosocial individuals reduce their contributions in light of the risk of exploitation; this spurs others to do likewise, creating a downward spiral (reviewed in Fehr and Schurtenberger 2018). More broadly, consonant with the adaptively relevant fact that dangers are often more imminent than, and preclude, opportunities, across many domains, negative events have greater attentional, emotional and motivational salience than positive events (Rozin and Royzman 2001; Baumeister *et al.* 2001). Accordingly, seeing an exemplary prosocial actor being exploited should markedly inhibit elevation elicitation.

To explore the above possibilities, in three experiments, we investigated the effects on elevation elicitation of social stimuli beyond those of a single exemplary prosocial actor. All study protocols reported in this paper were approved by the University of California, Los Angeles Office of the Human Research Protection Program. Informed consent was obtained before participation. Data and analysis code for all studies are at https://osf.io/6m2ya/.

## Study 1

### Study 1 methods

Based on results from our prior work (currently under review elsewhere), we targeted a sample size of 1800 (300 per condition). A total of 1804 US participants were recruited in April 2017 via Amazon Mechanical Turk (500+ completed HITs [the term Mechanical Turk uses for each paid assignment], 95% approval) in exchange for $1.20–1.30, depending on the length of the survey. Data were prescreened for repeat participation, minimal completeness, answering ‘catch questions’, excessively short completion time and technical problems reported by participants; see Supplementary Material for details. The final sample consisted of 1616 adults (54% female; 72.5% white), age 18–88 (*M* = 36.7, SD = 11.9).

In Study 1, we employed as a stimulus an edited version of *Unsung Hero*, a Thai television commercial depicting a young man engaging in various charitable acts toward strangers in his urban environment (e.g. giving money to a beggar; feeding a stray dog; leaving a gift of food for an elderly neighbor); recipients express gratitude toward their benefactor (e.g. a hug from the elderly neighbor; a smile from the beggar) and provide benefits to their benefactor (e.g. the dog assists the protagonist) (see Supplementary Material for all stimuli discussed in this paper). In a between-subjects design, participants in a control condition watched a video of a parkour athlete performing acrobatics in an urban environment – an entertaining (and arguably admirably exceptional) performance by a young man, but one lacking prosociality. As a second experimental condition, we edited the *Unsung Hero* video further, removing scenes of gratitude from and reciprocation by the protagonist's beneficiaries. As a matched control for this condition, we created an equivalently shortened parkour video. Note that, because acts of reciprocation are themselves prosocial, the shortened version of *Unsung Hero* contains fewer prosocial acts. Likewise, in the longer version multiple prosocial individuals are depicted (the protagonist and his reciprocating beneficiaries), but in the shorter version only a single prosocial individual (the protagonist) is shown. To examine the effects of the number of prosocial individuals, and number of prosocial acts, witnessed independent of the issue of reciprocity, using real-life videos collected from the Internet, we created a montage of video clips, each depicting a different individual engaged in one of a wide variety of prosocial acts (e.g. giving food to a beggar; inoculating poor children). As a control condition for witnessing multiple actors, we employed a video, of equivalent length, depicting a montage of neutral content featuring a similar variety of settings and people. Participants were randomly assigned to conditions.

In all conditions, participants first completed our highly face-valid self-report measure assessing idealism, the expectation that others will behave prosocially [e.g. ‘most people are basically honest’, ‘people cannot be good to each other’ (reverse coded); see Supplementary Material for all measures discussed herein]. They then watched an unremarkable 30 s video of commuters on a passenger train, then completed our self-report elevation scale which, resembling those used by prior elevation researchers, consists of items employing emotion terms (e.g. ‘inspired’, ‘uplifted’), somatic symptoms (e.g. ‘tears in eyes’), and behavioral tendencies (e.g. ‘be a good person’). This initial procedure is intended to place participants in a neutral emotional state and to familiarize them with our elevation scale. After several demographic questions (distracting from the aforementioned scale), participants watched the assigned video, then completed the elevation scale again, allowing for measurement of the effects of the stimulus video on emotional state.

### Study 1 results

**Figure 1.**
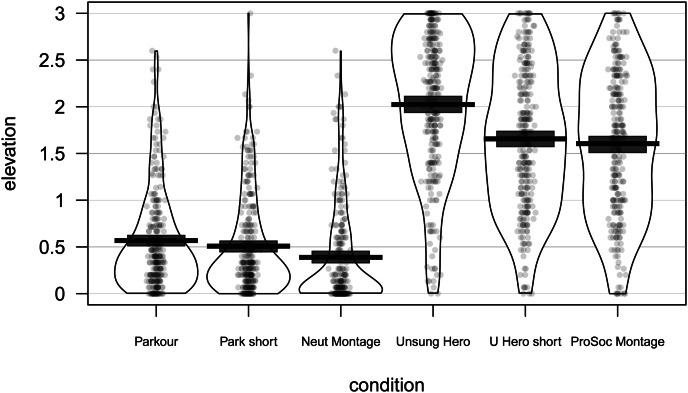
Elevation levels by condition in Study 1. Scatterplot points are raw data, jittered to reduce overlap. Beans show smoothed density of data points. Bars and boxes represent means and Bayesian 95% highest density intervals, respectively.

Our elevation scale was internally reliable (*α* = 0.97; see Supplementary Material for details). Elevation levels in each condition are visualized in Figure 1 (for the effects of condition on each subscale of the elevation measure, see Supplementary Material Figure S1). As anticipated, the control conditions (Neutral Montage, Parkour, Parkour Shortened) evince lower elevation levels than the prosocial conditions (Prosocial Montage, *Unsung Hero*, *Unsung Hero* Shortened): ∆*M* (difference in means) = −1.27, 95% CI [−1.33, −1.20], *t*(1, 480.50) = −38.26, *p* < 0.001 (see Supplementary Material for additional analyses). Among the prosocial conditions, *Unsung Hero* elicits more elevation than its shortened version or than the Prosocial Montage, with no significant difference between the latter two (Table 1). Our idealism scale was internally reliable (*α* = 0.93; see Supplementary Material for details). Idealism significantly interacted with condition type (prosocial vs control) to predict elevation (Supplementary Material Table S1) such that idealism was a significant predictor of elevation in all prosocial conditions and no control conditions (Supplementary Material Table S4).

**Table 1.** Linear regression model of elevation score as a function of condition, among prosocial conditions in Study 1

Predictor	*b*	95% CI	*t*(816)	*p*
Intercept	2.02	[1.93, 2.11]	44.13	<0.001
*Unsung Hero* shortened (dummy)	−0.37	[−0.49, −0.24]	−5.68	<0.001
Prosocial montage (dummy)	−0.42	[−0.55, −0.29]	−6.38	<0.001

Note: *Unsung Hero* is treated as reference group, with dummy variables for other conditions. Model fit: *F*(2, 816) = 24.56, *p* < 0.001, *R*^2^ = 0.06.

### Study 1 discussion

Per elementary predictions, in all three prosocial conditions, both in aggregate and by subscale, elevation was increased relative to that elicited in any of the control conditions. Likewise, per core predictions of our model, in all three prosocial conditions post-stimulus elevation correlated positively with pre-stimulus idealism. Addressing the key issue here, the experimental video depicting both prosociality and reciprocation seemed to elicit more elevation than either the same video edited to remove evidence of reciprocation or the montage video depicting prosocial acts by multiple individuals without reciprocation; the latter two stimuli elicited identical levels of elevation.

These results suggest that reciprocated prosocial acts may be more elevating than unreciprocated prosocial acts, consistent with the notion that observers are assessing not merely the presence of an exemplary prosocial actor, but also the milieu in which the payoffs for such actions do or do not occur. However, it is unclear whether the lesser elevation elicited by the shortened *Unsung Hero* video owes to the absence of reciprocation or instead derives from either the smaller number of prosocial acts depicted, the smaller number of prosocial individuals depicted, or both. The montage video was intended to test the importance of reciprocity, as it depicted multiple prosocial acts by multiple individuals, none of which involved reciprocation. However, it is difficult to conclude from the lack of difference between the montage condition and the shortened *Unsung Hero* condition that reciprocation is key, as the latter had higher production values and depicted a consistent narrative, such that the former was more taxing to watch, plausibly influencing elevation elicitation. Lastly, *Unsung Hero* and the parkour video were both accompanied by music; this soundtrack was slightly jumpy in the shortened version of *Unsung Hero*, while the prosocial montage contained no sound at all. Given the power of music to evoke strong emotions (Balteş *et al.* 2011), this inconsistency across conditions constituted a potential confound. We therefore conducted a second study in which we held the video content of the stimulus constant, and manipulated the information presented to participants using text at the end of the video.

## Study 2

### Study 2 methods

In Study 2, a final sample size of 600 was targeted (100 per condition) based on estimates derived from Study 1 results. A total 607 US participants were recruited in June of 2017, via Mechanical Turk as in Study 1, in exchange for $1.30. Exclusion criteria were the same as in Study 1; see Supplementary Material for details. The final sample consisted of 495 adults (55% female; 71.7% white), age 19–74 (*M* = 36.6, SD = 12.0); *post-hoc* analyses indicate that the power to detect an effect of the size observed in Study 1 using this sample was nearly 100% (see Supplementary Material).

To compare the effects on elevation of direct reciprocity, indirect reciprocity and a sole exemplary prosocial individual, in a between-subjects design, we employed the shortened version of the *Unsung Hero* video used in Study 1 followed by scrolling text that recounted either (a) direct reciprocation by the protagonist's beneficiaries (termed the Pay-It-Back condition) or (b) prosocial actions directed at third parties by his beneficiaries (termed the Pay-It-Forward condition). To control for the increased number of prosocial actions depicted, we created a third version in which the same video was followed by text recounting additional prosocial acts by the protagonist, but containing no information about his beneficiaries’ reactions (termed the Lone-Altruist condition). To explore whether, as predicted, elevation elicitation is diminished by observing exploitation, we created two conditions in which the text recounts exploitative responses from the protagonist's beneficiaries. In one (termed the Exploited condition), the text consists solely of accounts of this exploitation. However, because this condition contains a smaller total number of prosocial actions than in the Pay-It-Back, Pay-It-Forward and Lone-Altruist conditions, we also created a condition (termed the Martyr condition) in which the Exploited condition's accounts of exploitative responses are presented together with the descriptions of the protagonist's additional prosocial actions contained in the Lone-Altruist condition. Lastly, a quasi-control condition (termed the No-Additional-Information condition) was created by pairing the prosocial video with text providing no information about prosocial or antisocial acts. Participants were randomly assigned to conditions. Study 2 was pre-registered (see https://osf.io/vcpyg/).

### Study 2 results

**Figure 2.**
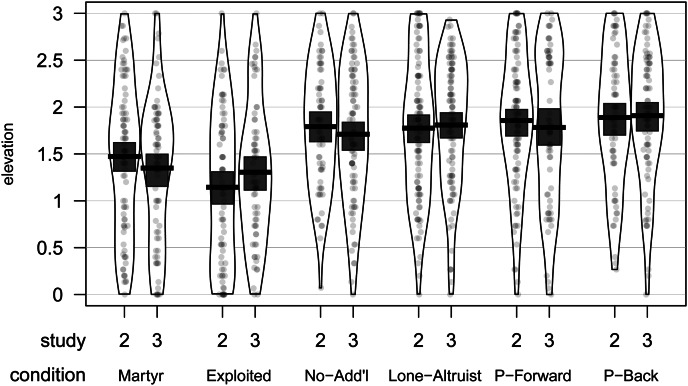
Elevation levels by condition and study, for Studies 2 and 3. Scatterplot points are raw data, jittered to reduce overlap. Beans show smoothed density of data points. Bars and boxes represent means and Bayesian 95% highest density intervals, respectively.

Our elevation scale was again internally reliable (*α* = 0.96; see Supplementary Material for details). Elevation levels in each condition are visualized in Figure 2 (for effects of condition on each elevation subscale, see Supplementary Material Figure S2). Among the conditions that include no evidence of antisociality, there are no significant differences in elevation levels F (3, 326) = 0.45, MSE (mean squared error) = 0.52, *p* = 0.719, 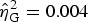 (see Supplementary Material for additional analyses). These conditions elicit more elevation than do those that include evidence of antisociality: ∆*M* = 0.51, 95% CI [0.36, 0.65], *t*(294.67) = 6.80, *p* < 0.001. Among the latter, the Martyr condition is more elevating than the Exploited condition: ∆*M* = 0.33, 95% CI [0.08, 0.57], *t*(160.79) = 2.64, *p* = 0.009. Our idealism scale was again internally reliable (*α* = 0.93; see Supplementary Material for details). Condition and idealism were significant predictors of elevation, but the interaction was not significant (Supplementary Material Table S2). Analyzing conditions separately, idealism significantly predicted elevation in four of the conditions (Supplementary Material Table S4).

### Study 2 discussion

In contrast to Study 1, Study 2 reveals no clear added effect of evidence of reciprocation, as the elevation elicited in the Pay-It-Back condition did not differ from that in the Lone-Altruist condition; nor was there a positive influence of indirect reciprocity, as the Pay-It-Forward condition likewise elicited essentially identical levels of elevation. However, consistent with predictions, there is clear evidence of the inhibitory effect of the presence of antisocial individuals on elevation elicitation, as both the Exploited condition and the Martyr condition produced less elevation than any of the purely prosocial conditions, with the Exploited condition being the least elevating.

*Ceteris paribus*, we might expect that, independent of the identity of the individuals responsible, additional evidence of prosocial actions should heighten elevation elicitation. However, in addition to there being no differences in elevation between the Pay-It-Back, Pay-It-Forward and Lone-Altruist conditions, these conditions all elicited the same level of elevation as the No-Additional-Information condition in which the text was uninformative regarding additional prosocial actions – resulting in a smaller total number of prosocial actions depicted. Given the lack of difference between the No-Additional-Information condition and the other prosocial conditions, it is possible that either participants did not attend fully to the text or else the video stimulus, with its greater realism, was sufficiently more evocative than the text that the information presented in the latter had little effect. Granted, the depressive effects of the Exploited and Martyr conditions on elevation indicate that the information presented in the text did register with participants. However, both general negativity bias and error-management considerations (Haselton and Nettle 2006) regarding the possibility of greater impacts on fitness of failing to detect cheaters relative to failing to detect cooperators (but see Delton *et al.* 2011) indicate that text recounting exploitation can be expected to have a greater absolute effect than text recounting additional prosocial acts and/or individuals. Hence, it is possible that text following the video has an effect on elevation, but this effect is more easily detected when it is negative than when it is positive, making the lack of difference between the positive conditions impossible to interpret. Finally, we note that participants’ previous familiarity with the *Unsung Hero* video (widely viewed on the Internet) could have reduced the effectiveness of the altered endings, yet we failed to measure this.

## Study 3

### Study 3 methods

In Study 3, a final sample size of 600 was again targeted (100 per condition). A total of 604 US participants were recruited in July 2018, via Mechanical Turk as in Study 1, in exchange for $1.30. Exclusion criteria were the same as in Study 1. The final sample consisted of 476 adults (48% female; 72.7% white), age 18–76 (*M* = 35.4, SD = 11.4).

To address Study 2's limitations, we replicated the Study 2 design, substituting text accounts of the story depicted in the video and melding this with the texts that followed the video in the various conditions of Study 2 (participants were again randomly assigned to conditions). While this sacrifices the evocative power of video, by muting the contrast between the initial depiction of prosociality and the manipulations that follow, we obtain a clearer test of whether said manipulations influence the elicitation of elevation. Additionally, we altered superficial details of the story to mask similarity to *Unsung Hero* in order to reduce the likelihood that previous familiarity with *Unsung Hero* colors participants’ interpretation of the narrative; in addition, following completion of dependent measures, participants were queried as to their familiarity with *Unsung Hero*. Lastly, we used a slightly refined version of our idealism scale (see Supplementary Material). All other methods were identical to those of Study 2. Study 3 was pre-registered (see https://osf.io/dn6wk/).

### Study 3 results

Our elevation scale was once more reliable (*α* = 0.95; see Supplementary Material for details). Elevation levels in each condition are visualized in Figure 2 (for effects of condition on elevation subscales, see Supplementary Material Figure S2). Once again, the conditions that do not include evidence of antisociality elicit more elevation than the conditions that do: ∆*M* = 0.47, 95% CI [0.33, 0.62], *t*(266.38) = 6.40, *p* < 0.001. Replicating Study 2, there are no differences in elevation among the four conditions lacking evidence of antisociality: *F*(3, 328) = 1.05, MSE = 0.53, *p* = 0.372, 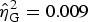 (see Supplementary Material for additional analyses). Unlike Study 2, the two conditions containing evidence of antisociality do not significantly differ from one another: ∆*M* = 0.04, 95% CI [−0.20, 0.29], *t*(141.74) = 0.35, *p* = 0.726. Our idealism scale was again internally reliable (*α* = 0.82; see Supplementary Material for details). Condition and idealism significantly predicted elevation, but the interaction was not significant (Supplementary Material Table S3); idealism predicted elevation in all conditions (Supplementary Material Table S4). Lastly, in an ANOVA modeling elevation as a function of condition, previous familiarity with Unsung Hero and their interaction, we find no significant interaction, and main effects of condition and having previously viewed the *Unsung Hero* video. Non-naive participants reported higher elevation (see Supplementary Material Tables 2 and 3 and Supplementary Material Figure S3). This is unlikely to be due to self-selection for previous viewing, as idealism does not predict prior viewing (see Supplementary Material).

### Study 3 discussion

Presumably reflecting the lower evocative power of our text accounts relative to professionally produced videos, responses across subscales (particularly in regard to somatic items) are slightly depressed in Study 3 compared with Studies 1 and 2 (see Supplementary Material Figure S2). Despite this minor difference, overall, Study 3 replicated the results of Study 2 – once again there are no significant differences between the conditions that exclusively portray prosocial behavior, suggesting that, within the confines of our experimental paradigm, elevation elicitation is insensitive to evidence of either direct or indirect reciprocity, nor is it influenced by evidence of larger numbers of either prosocial acts or prosocial actors. In contrast, robustly replicating our prior findings, accounts of prosocial behavior being met by exploitation markedly erode the elicitation of elevation. Unlike Study 2, we find no difference between a depiction of an actor who persists in being prosocial in the face of exploitation and a depiction of an actor whose various altruistic acts are collectively followed by exploitation – suggesting that the salient feature is the presence of exploitation, not prosocial responses to exploitation. Prior familiarity with *Unsung Hero* predicts greater elevation across conditions, suggesting that our attempts to mask the source of our textual stimuli were incompletely successful. Given evidence suggesting that such prior familiarity, a likely methodological confound, inflates elevation scores more in the conditions in which exploitation occurs (see Supplementary Material Figure S3), the depressive effects of the exploitation on elevation may be even stronger than our results indicate.

## General discussion

Across three studies, we find that, consonant with our core model, baseline idealism generally predicts the experience of elevation in response to prosocial stimuli, indicating that, per the hypothesized mechanism, the propensity to upregulate prosocial motives after having observed prosocial behavior is contingent on prior expectations regarding the likelihood that others will act prosocially. Our model is premised on the insight that, when attempting to forecast whether behaving prosocially will be profitable, there is always uncertainty in interpreting limited observations of others’ actions, hence it pays to weigh these observations in light of past experience. Similar considerations led us to predict that observing the beneficiaries of prosocial behavior acting in kind, either by reciprocating toward their benefactor or by benefiting others, would enhance the elicitation of elevation, as seeing multiple others behaving prosocially provides additional information as to the likely payoffs for the witness who responds with an emotion driving prosociality. However, despite observing some support for this prediction in Study 1, our overall results indicate that elevation elicitation appears not to be influenced by these factors, nor is it affected by the simple dimensions of number of prosocial acts, or of prosocial actors, observed.

At least four possible explanations apply. First, if elevation does not serve the adaptive function that we have sketched, then predictions derived from this model will generally fail. While we cannot rule this out, the nature of the relationship between idealism and elevation – predicted *a priori* by, and exclusive to, our account, and repeatedly supported here and elsewhere – militates against this. Second, it is possible that our core model is correct, but that we have underestimated the importance of the presence of a single exemplary prosocial actor. Perhaps, if the focal individual's actions are sufficiently beneficial to others and sufficiently consistent over the observed period, information concerning others’ prosocial behavior adds little to the assessed profitability of prosociality, as the observer who upregulates prosociality will ultimately benefit from the focal individual through direct or indirect reciprocity. Third, it is possible that the aforementioned results reflect methodological limitations. Emotions elicited by brief videos or shallow text descriptions are necessarily weak echoes of those experienced in real situations. Gradations of elevation that would be evident in responses to actual events may therefore be compressed in our results, to the point that they are unobservable. That witnessing exploitation produces measurably different results using the same methods need not vitiate this explanation, as, owing to error management and negativity bias, the absolute effects of cues of exploitation on elevation elicitation may be much greater than the effects of cues of the presence of directly or indirectly reciprocating beneficiaries of prosociality, and hence such corrosive effects may be evident even in artificial contexts such as our experiments. Fourth, participants’ responses may in part reflect the real social interaction in which they are engaged – interacting with the experimenters who are employing them to experience a pleasing video – rather than being exclusively driven by the fictitious stimuli. Presenting a video depicting prosociality, compared with sharing a merely entertaining video, might be more likely to be regarded as an invitation to cooperate or an attempt to manipulate; some participants’ emotions and cooperative motives could be at least partially directed towards the experimenter who ‘introduced’ them to the characters in the video. It is unclear if or how variation in the details of the cooperative narrative might influence the participant's relationship with the researchers.

Consonant with predictions, observing antisocial responses markedly diminishes elevation elicited by an exemplary prosocial actor. Whether because exploiters (a) provide contrasting information about the immediate prevalence of prosociality; (b) themselves pose a threat to an observer who engages in increased prosociality; or (c) impair the prosocial actor’s ability to reward the observer for prosociality, or for all of these reasons, the presence of antisocial actors reduces the expected payoffs of prosociality, and thus should diminish elevation elicitation. That this diminution indeed occurs, and yet is not absolute, underlines the power of a single exemplary prosocial actor to elicit elevation. In exploring these dynamics, a key question for future research will be to determine whether, on the one hand, diminished elevation occurs because antisociality elicits a negatively-valenced emotion, such as moral outrage or moral disgust (see Haidt 2000), that subserves the punishment of antisocial others and competes with elevation, or, on the other hand, observing antisociality exercises a direct depressive effect on elevation elicitation. Either way, tempering elevation elicitation in the presence of exploitation is consonant with our central thesis that the function of elevation is to adjust the motivation to behave prosocially in light of the assessed profitability of such actions in the current context.

Unlike in Study 2, in Study 3, we find that the corrosive effects on elevation elicitation of witnessing exploitation are independent of whether the prosocial protagonist persists in providing benefits in the face of abuse. If the null effect is more reliable, this would suggest that martyrs who sacrifice for others while suffering their depredations do not hold unique evocative power in regard to elevation. History is replete with celebrated prosocial martyrs, hence the latter finding may reflect the skeletal nature of our depictions of reactions to suffering exploitation. However, it is also possible that detailed depictions of such martyrs’ sacrifices will elicit emotions that overlap with, but are not isomorphic with, elevation. If elevation serves to adjust prosocial motivation in light of the assessed immediate profitability of prosociality, then exploited martyrs may inspire admiration rather than elevation, since exploitation remains a deterrent to upregulating prosociality independent of the martyr's actions.

Although here we have operationalized idealism only in the most generic terms (e.g. ‘most people are basically honest’, etc.) our overarching model suggests that individuals probably hold not one attitude of idealism/cynicism, but many, each specific to a given community or social category. In combination with idealism's influence on elevation elicitation and subsequent contagious transmission of prosociality, this potentially illuminates how multiple social equilibria can occur, such that groups or communities exist across the spectrum from highly prosocial to highly antisocial. Relatedly, here we have conceptualized attitudes in an artificially narrow sense. If attitudes are representations of the fitness affordances of others for the observer, then what we have termed idealism is necessarily a gross simplification, as actual attitudes should also contain information about whether the observer would be accepted as a prosocial partner by members of the specified group or category, whether the group's aims align or conflict with the observer's goals, etc. In future research it will be important to move beyond measurements of idealism writ large, and instead explore how more fully specified attitudes predict elevation in response to observed behaviors. Likewise, the present work relies exclusively on US Mechanical Turk participants; given likely cultural variation in both broad and specific idealisms, and expectable cultural variation in responses to antisociality (Leung and Cohen 2011), in the future it will be vital to conduct this research across cultures and subcultures. Lastly, our findings suggest that interventions intended to shift the equilibrium toward greater prosociality must be carefully designed and deployed, as the elicitation of elevation, and thus the sparking of virtuous cycles of increased prosociality, may at best be handicapped, and at worst precluded, if individuals modeling marked prosociality are exploited by others in the community.

## Author contributions

D.F. and T.S. conceived the project, with input from A.S. and C.H. T.S. created study materials with input from D.F., A.S. and C.H., and oversaw data collection. A.S. conducted the analyses. D.F. wrote the manuscript with principal input from A.S. and additional input from C.H. and T.S.

## Supplementary material

For supplementary material accompanying this paper visit http://dx.doi.org/10.1017/ehs.2019.3. click here to view supplementary material
