# Supplementary material for: Does observing reciprocity or exploitation affect elevation, a mechanism driving prosociality?
Source: Evol Hum Sci. 2019 May 23;1:e3. doi: 10.1017/ehs.2019.3 (PMC10427316; doi:10.1017/ehs.2019.3)
Supplement: Supplementary file 1 [file S2513843X19000033sup001.pdf]

# Supplement | Does Observed Reciprocity or Exploitation Affect Elevation, a Mechanism Driving Prosociality?

Daniel M.T. Fessler et al[1]

[1] UCLA

Author Note

Correspondence concerning this article should be addressed to Daniel M.T. Fessler et al, UCLA Anthropology. E-mail: dfessler@anthro.ucla.edu

Abstract . . . 3

**Additional Results** **4**

Elevation subscales and positive affect . . . 4

Correlations between idealism and elevation . . . 7

Does previous familiarity with Unsung Hero video matter? . . . 11

Sex Differences . . . 13

**Methods Details** **17**

Study 1 . . . 17

Study 2 . . . 22

Study 3 . . . 26

Scale Reliabilities . . . 33

Power Analysis and Sample Size . . . 34

Elimination decisions . . . 34

## Abstract

Fitness is enhanced by determining when to behave prosocially. *Elevation,* an uplifting emotion elicited by witnessing exemplary prosociality, upregulates prosociality in the presence of prosocial others, as such contexts render prosociality profitable and/or antisociality costly. Prior research examines responses to a single highly prosocial individual. However, the profitability of enhancing prosociality hinges not only on potential interactions with a single actor, but also on the actions of others. Accordingly, information regarding how others respond to the prosocial exemplar may influence elevation elicitation and corresponding changes in prosocial motivation. If others reciprocate the exemplar's prosociality, or pay prosociality forward, this expands opportunities for the observer to profit by increasing prosociality, and thus could enhance elevation elicitation. Conversely, if others exploit the exemplar, this may diminish the profitability of prosociality, as the observer who acts prosocially may similarly be exploited and/or the resources with which the exemplar could reciprocate will be depleted. Conducting three online studies of Americans in which information regarding the responses of others to a prosocial exemplar was manipulated, we find that, against predictions, prosocial responses by the beneficiaries of prosociality generally do not enhance elevation among observers, whereas, consonant with predictions, antisocial responses markedly diminish elevation among observers.

# Additional Results

## Elevation subscales and positive affect

In main text, we report elevation levels in each condition of each study. For similar information for elevation's three subscales (somatic symptoms, folk affect terms, and prosocial motives) and a measure of positive affect, see Figures 1 & 2.

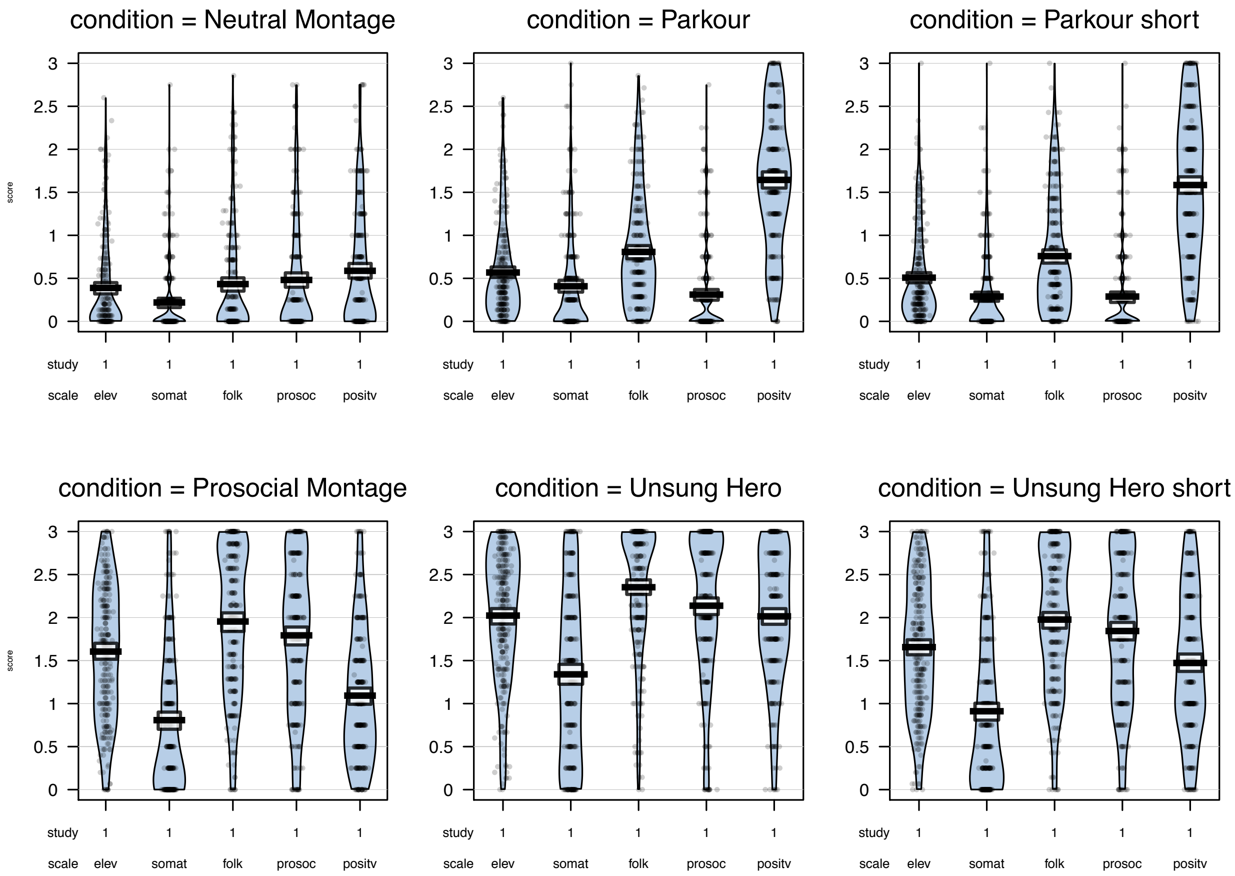


*Figure 1*. Elevation, subscale, and positive affect by condition, Study 1. Scatterplot points are raw data, jittered to reduce overlap. Beans show smoothed density of data points. Bars and boxes represent means and Bayesian 95% highest density intervals, respectively. Scales represented are the overall elevation scale (elev), somatic subscale (somat), folk affect terms subscale (folk), prosocial motives subscale (prosoc) and the positive affect scale (positv).

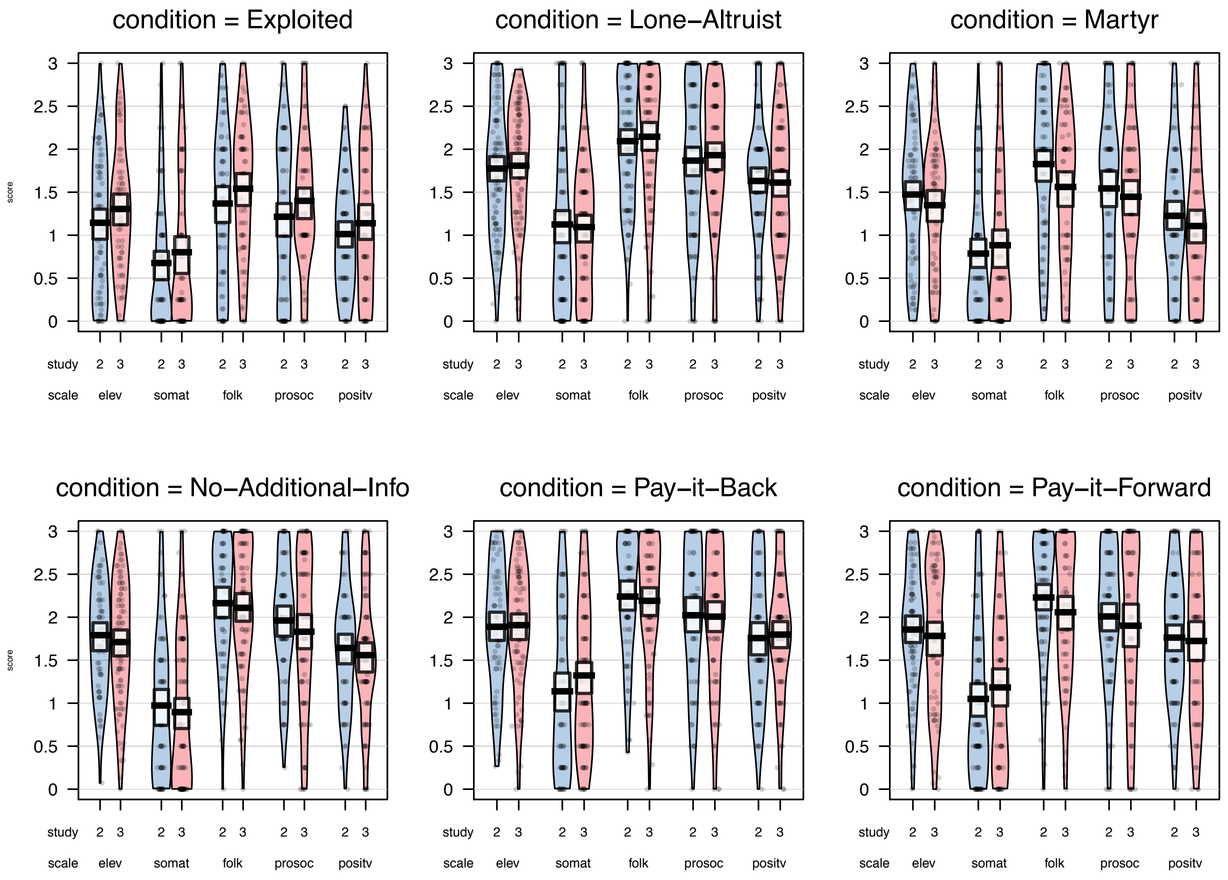


*Figure 2.* Elevation, subscale, and positive affect by condition, Study 2 & 3. Scatterplot points are raw data, jittered to reduce overlap. Beans show smoothed density of data points. Bars and boxes represent means and Bayesian 95% highest density intervals, respectively. Scales represented are the overall elevation scale (elev), somatic subscale (somat), folk affect terms subscale (folk), prosocial motives subscale (prosoc) and the positive affect scale (positv).

Table 1

*ANOVA model of elevation score as a function of condition type (prosocial versus control) and idealism, in Study 1.*

| Effect | $F$ | $df_1$ | $df_2$ | $MSE$ | $p$ | $\hat{\eta}^2_G$ |
|---|---|---|---|---|---|---|
| Prosocial | 1,171.47 | 1 | 1341 | 0.47 | $< .001$ | .466 |
| Idealism | 37.22 | 1 | 1341 | 0.47 | $< .001$ | .027 |
| Prosocial × Idealism | 7.57 | 1 | 1341 | 0.47 | .006 | .006 |

## Correlations between idealism and elevation

In previous work (Sparks et al, in review) we have reported that idealism predicts elevation in response to a prosocial video (Unsung Hero) but not control videos. The relationship between idealism and elevation in each condition of the current three studies is reported in Table 4.

Table 2

*ANOVA model of elevation score as a function of condition type (prosocial versus control) and idealism, in Study 2.*

| Effect | $F$ | $df_1$ | $df_2$ | $MSE$ | $p$ | $\hat{\eta}^2_G$ |
|---|---|---|---|---|---|---|
| Condition | 12.63 | 5 | 483 | 0.53 | $<$ .001 | .116 |
| Idealism | 28.76 | 1 | 483 | 0.53 | $<$ .001 | .056 |
| Condition $\times$ Idealism | 0.86 | 5 | 483 | 0.53 | .508 | .009 |

In Study 1, idealism significantly interacted with condition type (prosocial versus control) to predict elevation (Table 1) such that idealism was a significant predictor of elevation in all prosocial conditions and no control conditions (Table 4). Note that the idealism measure was accidentally dropped from the Parkour condition and that Study 1's Unsung Hero condition was included in the meta-analyses reported by Sparks et al.

Table 3

*ANOVA model of elevation score as a function of condition type (prosocial versus control) and idealism, in Study 3.*

| Effect | $F$ | $df_1$ | $df_2$ | $MSE$ | $p$ | $\hat{\eta}^2_G$ |
|---|---|---|---|---|---|---|
| Condition | 9.97 | 5 | 464 | 0.49 | $< .001$ | .097 |
| Idealism | 56.73 | 1 | 464 | 0.49 | $< .001$ | .109 |
| Condition × Idealism | 0.27 | 5 | 464 | 0.49 | .932 | .003 |

In Study 2, condition and idealism were significant predictors of elevation, but the interaction was not significant (Table 2). Analyzing conditions separately, idealism significantly predicted elevation in four of the conditions; the exceptions were the Pay-it-Back and Martyr conditions (Table 4). In Study 3 condition and idealism again were significant predictors of elevation, but the interaction was not significant (Table 3); idealism predicts elevation in all conditions (Table 4).

Table 4

*Correlations between idealism and elevation, by study and condition*

| study | condition | Correlation [CIs] | df | p |
|---|---|---|---|---|
| Study 1 | Prosocial Montage | .28 [.17 .39] | 262 | < .001 |
| Study 1 | Neutral Montage | .10 [-.03 .22] | 247 | .119 |
| Study 1 | Unsung Hero | .19 [.07 .30] | 274 | .002 |
| Study 1 | Unsung Hero short | .15 [.03 .26] | 277 | .012 |
| Study 1 | Parkour short | .08 [-.04 .20] | 275 | .172 |
| Study 2 | Lone-Altruist | .33 [.14 .49] | 100 | .001 |
| Study 2 | Pay-it-Back | .07 [-.17 .30] | 67 | .572 |
| Study 2 | No-Additional-Info | .30 [.06 .51] | 63 | .015 |
| Study 2 | Pay-it-Forward | .24 [.04 .42] | 92 | .020 |
| Study 2 | Martyr | .16 [-.05 .36] | 85 | .135 |
| Study 2 | Exploited | .30 [.09 .49] | 76 | .007 |
| Study 3 | No-Additional-Info | .28 [.07 .46] | 84 | .009 |
| Study 3 | Lone-Altruist | .43 [.25 .58] | 95 | < .001 |
| Study 3 | Exploited | .40 [.18 .58] | 67 | .001 |
| Study 3 | Martyr | .23 [.01 .44] | 73 | .043 |
| Study 3 | Pay-it-Forward | .34 [.11 .54] | 64 | .005 |
| Study 3 | Pay-it-Back | .27 [.06 .46] | 81 | .013 |

Table 5

*ANOVA model of elevation score as a function of condition and previous familiarity with Unsung Hero in Study 3.*

| Effect | $F$ | $df_1$ | $df_2$ | $MSE$ | $p$ | $\hat{\eta}^2_G$ |
|---|---|---|---|---|---|---|
| Condition | 8.90 | 5 | 461 | 0.54 | $< .001$ | .088 |
| Seen before | 6.74 | 1 | 461 | 0.54 | .010 | .014 |
| Condition × Seen before | 0.89 | 5 | 461 | 0.54 | .490 | .010 |

**Does previous familiarity with Unsung Hero video matter?**

Participants who had previously seen the Unsung Hero video might differ from naive viewers in at least two ways. First, idealists might be more likely to seek out and/or watch this type of video. Second, our efforts to experimentally alter the story details might have unpredictable effects on those who have previously seen it. In (only) Study 3, participants indicated if they had seen the Unsung Hero video.

There is no indication that previous familiarity with the video is related to idealism; if anything, naive viewers tended to be slightly more idealistic: $\Delta M = 0.14$, 95% CI $[-0.09, 0.36]$, $t(180.63) = 1.18$, $p = .238$.

Figure 3 depicts the pattern of elevation responses by condition, split based on previous familiarity with the video. An ANOVA with condition, previous familiarity, and their interaction as predictors of elevation (Table 5) indicates that both condition and previous familiarity with the video are significant factors, but their interaction is not. An alternative model substituting condition valence (negative vs non-negative) for condition (Table 6) also finds condition valence and previous familiarity to be significant predictors, and the interaction between these is a marginally insignificant predictor.

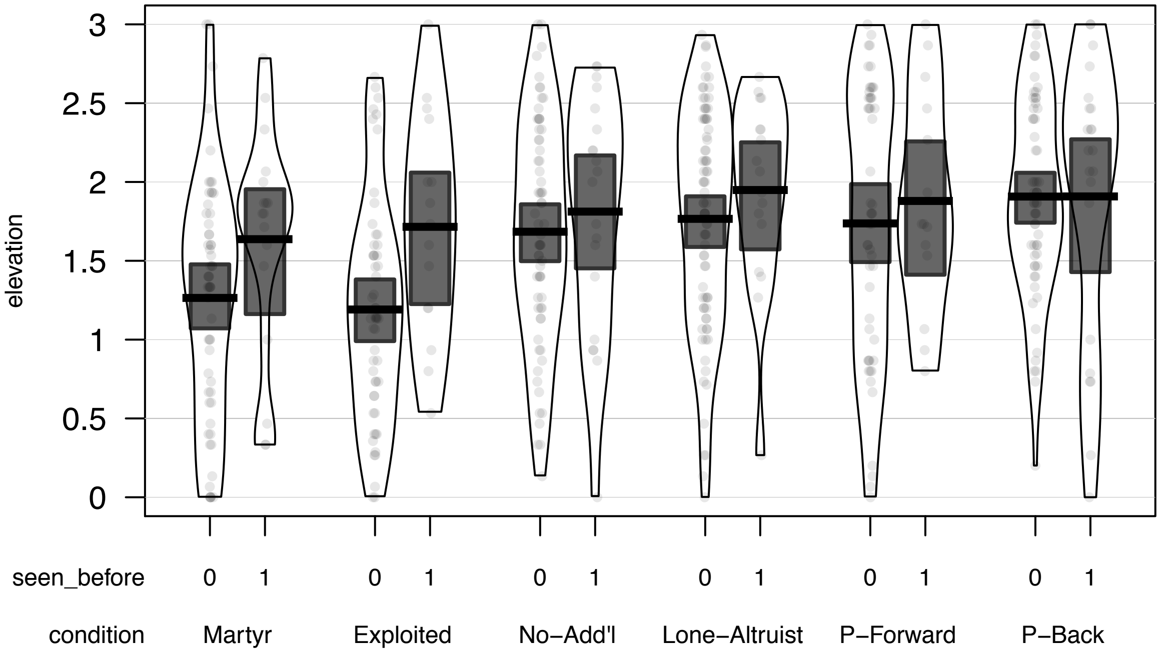


*Figure 3*. Elevation levels by condition, split by previous familiarity with the Unsung Hero video. There is a main effect whereby those who had previously seen the video (seen_before = 1) report higher elevation levels. There is weak evidence that this effect may be stronger for the negative conditions.

In summary, there is a main effect whereby those who have previously seen Unsung Hero report more elevation than those who have not. There is no evidence that these two groups differ in idealism. There is no strong evidence that the effect of experience with the video differs by condition, although there are trends hinting that the negative conditions may have stronger effects.

Table 6

*ANOVA model of elevation score as a function of condition valence (negative or non-negative) and previous familiarity with Unsung Hero in Study 3.*

| Effect | $F$ | $df_1$ | $df_2$ | $MSE$ | $p$ | $\hat{\eta}^2_G$ |
|---|---|---|---|---|---|---|
| Negative | 41.67 | 1 | 469 | 0.53 | $< .001$ | .082 |
| Seen before | 7.03 | 1 | 469 | 0.53 | .008 | .015 |
| Negative × Seen before | 3.59 | 1 | 469 | 0.53 | .059 | .008 |

## Sex Differences

We generally observe minor sex differences, with women reporting slightly higher levels of elevation than men in prosocial conditions. See Tables 7 - 9 and Figures 4 - 6.

Table 7

*ANOVA table for Study 1.*

| Effect | $F$ | $df_1$ | $df_2$ | $MSE$ | $p$ | $\hat{\eta}^2_G$ |
|---|---|---|---|---|---|---|
| Condition | 324.99 | 5 | 1592 | 0.42 | $< .001$ | .505 |
| Sex | 10.22 | 1 | 1592 | 0.42 | .001 | .006 |
| Condition × Sex | 7.16 | 5 | 1592 | 0.42 | $< .001$ | .022 |

Table 8

*ANOVA table for Study 2.*

| Effect | $F$ | $df_1$ | $df_2$ | $MSE$ | $p$ | $\hat{\eta}^2_G$ |
|---|---|---|---|---|---|---|
| Condition | 12.64 | 5 | 480 | 0.54 | $< .001$ | .116 |
| Sex | 16.74 | 1 | 480 | 0.54 | $< .001$ | .034 |
| Condition × Sex | 0.27 | 5 | 480 | 0.54 | .932 | .003 |

Table 9

*ANOVA table for Study 3.*

| Effect | $F$ | $df_1$ | $df_2$ | $MSE$ | $p$ | $\hat{\eta}^2_G$ |
|---|---|---|---|---|---|---|
| Condition | 9.45 | 5 | 459 | 0.53 | $< .001$ | .093 |
| Sex | 9.13 | 1 | 459 | 0.53 | .003 | .020 |
| Condition × Sex | 0.59 | 5 | 459 | 0.53 | .708 | .006 |

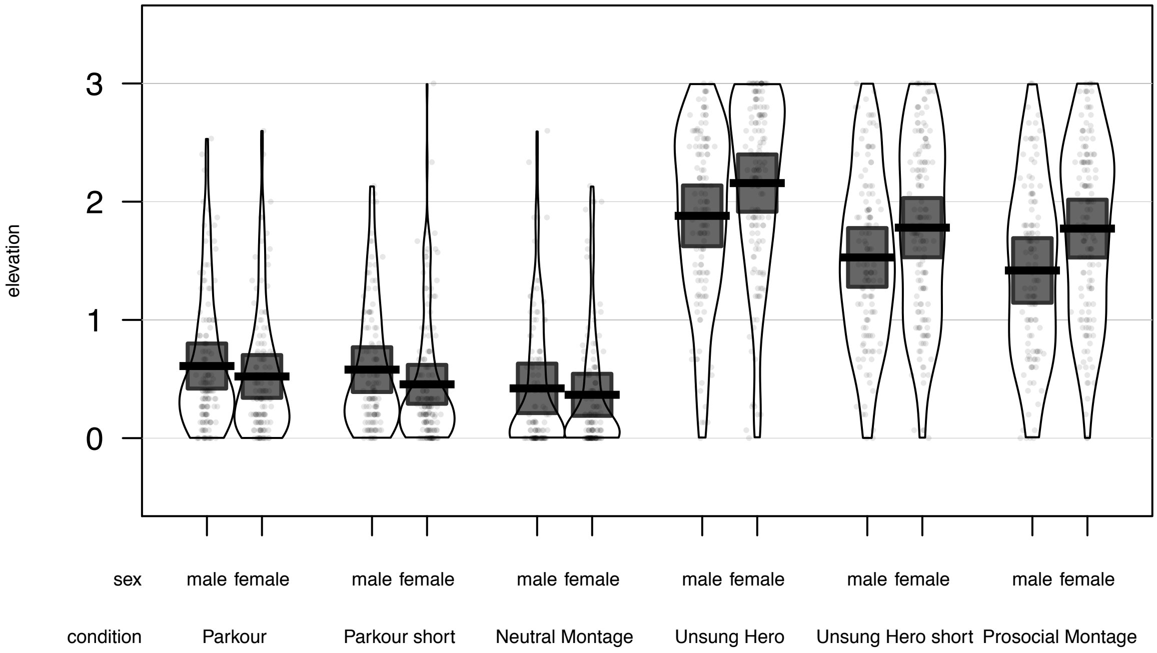


*Figure 4.* Sex differences in Study 1

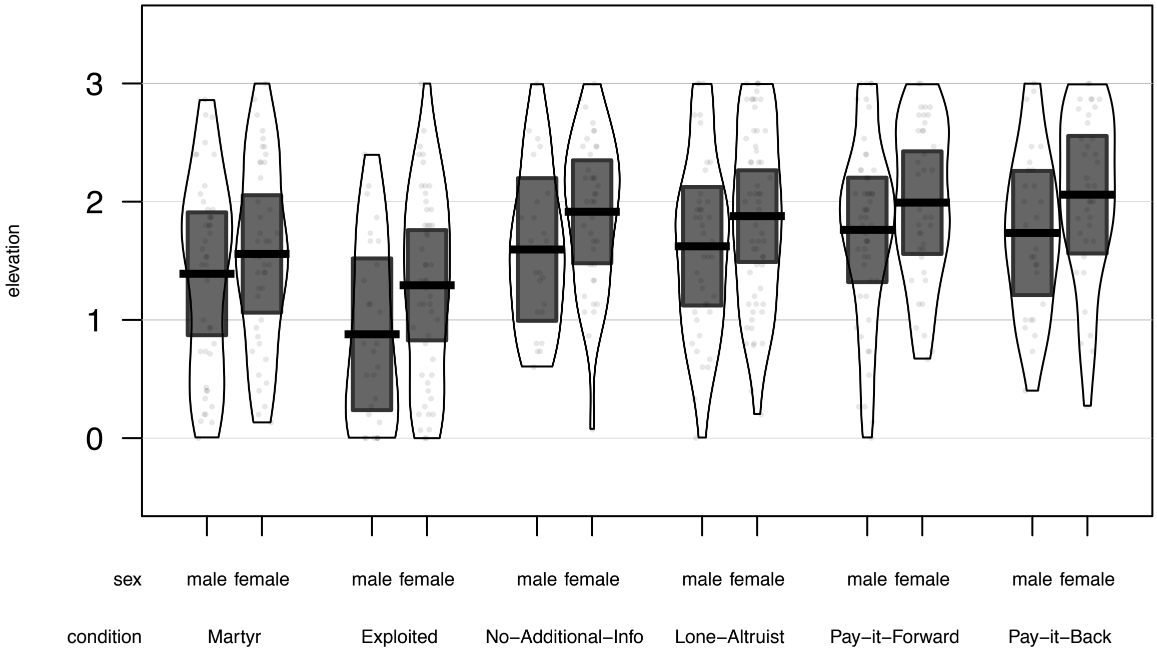


*Figure 5*. Sex differences in Study 2

.

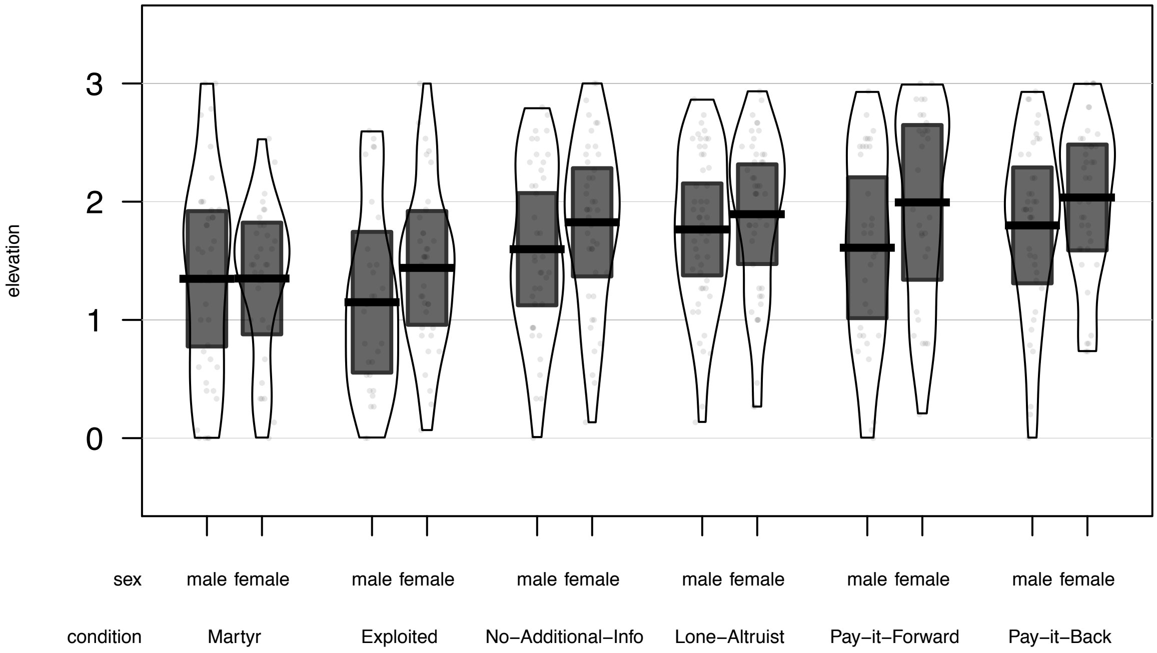


*Figure 6*. Sex differences in Study 3

## Methods Details

### Study 1

1. Idealism Scale (Sparks et al., n.d.)

Instructions: Please rate your level of agreement with the following statements about people in general. 1-strongly disagree to 7-strongly agree

Items:

- People don't try to be fair
- Most people are not trustworthy

- People try to be helpful
- If in doubt, I trust others
- The actions of most people are often admirable
- Most people are basically good-natured and kind
- Most people trust others
- I am optimistic about humanity
- Most people care about more than just themselves
- People are just looking out for themselves
- People can be good to each other
- People try to take advantage of you if they get the chance
- Most people are basically dishonest
- There's is very little good in the world
- You can't be too careful in dealing with people

2. Presentation of an affectively neutral video: [https://www.youtube.com/embed-/HbZZ0iJx-fE?rel=0&showinfo=0]

3. Elevation Scale (Sparks et al., n.d.) (listed here by subscale for purposes of exposition only; in actual presentation, no subscale headings appear, and items from all subscales are interspersed)

   Instructions: How much did the passage make you feel… [0-not at all to 3-strongly]

   Items:

   Emotion terms subscale:

- Uplifted
- Heartwarming feeling (in the metaphorical sense)
- Compassion

- Admiration
- Touched
- Moved
- Inspired

Behavioral tendencies subscale:

- Feel close to other people
- Want to help
- Want to be better person
- Want to find new ways to help

Somatic symptoms subscale:

- Tears in eyes
- Lump in throat
- The physical sensation of warmth in the chest
- Goose bumps / chills / tingles

Positive Affect scale:

- Happy
- Amused
- Entertained
- Delighted

4. Demographics 1

Items:

- Gender
- Age

- Parental status
- Height
- Type of device used to complete survey

5. Presentation of affectively positive video

   Conditions:

- Unsung Hero (prosocial narrative): https://www.youtube.com/embed/BJIExFjyvyI?rel=0&showinfo=0
- Parkour video (control): https://www.youtube.com/embed/Mhh66ufIG30?rel=0&showinfo=0
- Unsung Hero, short (prosocial narrative without reciprocity): https://www.youtube.com/embed/QpVQQeHgmtY?rel=0&showinfo=0
- Parkour video, short (control): https://www.youtube.com/embed/DBqSqitAx8w?rel=0&showinfo=0
- Prosocial Montage: https://www.youtube.com/embed/bmVO8HEUM9E?rel=0&showinfo=0
- Control Montage: https://www.youtube.com/embed/KrUJeM1Eru0?rel=0&showinfo=0

6. Elevation Scale (same as previous)

7. Study Checks

   Items:

- Sound problems
- Playback problems
- Attention check (If you go outside on a clear day and look up, what color should the sky usually be?)

8. Demographics 2

   Items:

- Political orientation (very liberal to very conservative)
- Ethnicity
- Yearly income
- Highest level of education completed

**Study 2**

1. Idealism Scale (see Study 1)

2. Presentation of an affectively neutral video (see Study 1)

3. Elevation Scale (see Study 1)

4. Demographics 1 (see Study 1)

5. Presentation of hybrid video/text stimuli (participants in all conditions watch the shortened version of the Unsung Hero video used in Study 1, which depicts the protagonist's prosocial actions, but does not depict the responses of those whom he benefits. The video is then followed by one of six text-based vignette endings that continue the narrative.)

   Vignette endings:

- Pay-It-Back condition (direct reciprocation by the beneficiaries) [https://www.youtube.com/embed/mjyjAYhZry4?rel=0&showinfo=0]

*In the weeks that followed, several things happened to the young man. While cooking dinner, he sat down to rest, and, tired from a long day, fell asleep. He was awakened by the dog barking and pawing at him, and realized that the food on the stove had caught fire; he put out the fire just in time. While helping the street vendor move her cart, he strained his back. Every evening she brought him food, and arranged for her brother to give him medication and hot compresses; he soon recovered. The old woman who lived next door observed that he accidentally left his door ajar when leaving for work; she locked the door and closed it for him. Lastly, on payday, after he had paid for his lunch at the cafe, his wallet fell out of his pocket. The homeless girl, sitting with her mother across the street, saw what happened; she ran over, picked up the wallet, caught up with him, and returned it to him.*

- Pay-It-Forward condition (prosocial actions directed at third parties by the beneficiaries) [https://www.youtube.com/embed/-SNaF-dtWGI?rel=0&showinfo=0]

*In the weeks that followed, several things happened in the young man's neighborhood. While preparing food, the cook sat down to rest, and, tired from a long day, fell asleep. He was awakened by the dog barking and pawing at him, and realized that the food on the stove had caught fire; he put out the fire just in time. While moving some boxes, the shopkeeper strained his back. The street vendor brought him food every evening, and arranged for her brother to give him medication and hot compresses; he soon recovered. The young man's elderly neighbor observed that another resident of the building accidentally left his door ajar when leaving for work; she locked the door and closed it for him. Lastly, on payday, after he paid for his lunch at the cafe, a stranger accidentally forgot his wallet. The homeless girl, sitting with her mother across the street, saw what happened; she ran over, picked up the wallet, caught up with the man, and returned it to him.*

- Lone-Altruist condition (additional prosocial acts by the protagonist) [https://www.youtube.com/embed/p7_4NnzKX8o?rel=0&showinfo=0]

*In the weeks that followed, several things happened in the young man's neighborhood. While preparing food, the cook sat down to rest, and, tired from a long day, fell asleep. While the cook was asleep, his stove caught fire; the young man, passing by, saw the flames and put out the fire just in time. While moving some boxes, the shopkeeper strained his back. The young man brought him food every evening, and gave him medication and hot compresses; he soon recovered. The young man observed that the elderly neighbor accidentally left her door ajar when leaving for work; he locked the door and closed it for her. Lastly, a stranger was eating lunch at the cafe on payday; after the stranger paid for his lunch, he accidentally forgot his wallet. The young man saw what happened, picked up the wallet, caught up with the stranger, and returned it to him.*

- Exploited condition (protagonist exploited by his beneficiaries) [https://www.youtube.com/embed/zAbdqdlyX4U?rel=0&showinfo=0]

*In the weeks that followed, several things happened to the young man. He came home to find that the dog had run away. While he was helping the street vendor move her cart, he twisted his ankle painfully. The street vendor rolled her eyes and kept pushing the cart down the road. The old woman who lived next door observed that he accidentally left his door ajar when leaving for work; she snuck inside and stole some fruit from his pantry. Lastly, on payday, while he was walking down the street, his wallet fell out of his pocket. The young homeless girl noticed but said nothing; when he wasn't looking, she quickly picked up his wallet and put it in her pocket.*

- Martyr condition (protagonist continues acting prosocially following exploitation by his beneficiaries) [https://www.youtube.com/embed/x9Wj__Fl8KrA?rel=0&showinfo=0]

*In the weeks that followed, several things happened to the young man. He came home to find that the dog had run away. While he was helping the street vendor move her cart, he twisted his ankle painfully. The street vendor rolled her eyes and kept pushing the cart down the road. The old woman who lived next door observed that he accidentally left his door ajar when leaving for work; she snuck inside, and the young man returned and caught her stealing his fruit. Lastly, on payday, his wallet fell out of his pocket. The young homeless girl saw this, and when she thought he wasn't looking, she quickly picked up his wallet and put it in her pocket. The young man noticed but said nothing.*

*Later, when moving her cart, the street vendor strained her back. Every evening the young man brought her food, and arranged for his brother to give her medication and hot compresses; she soon recovered. The young man noticed that he hadn't seen his elderly neighbor all day. Worried, he went into her apartment and saw her passed out, slumped in a chair. He rushed her to the hospital, and later, after discovering she had had a stroke, paid*

*for her medical bills. Finally, when he saw the young homeless girl and her mother begging on the street, he put money in their cup.*

*No-Additional-Information condition (no further prosocial or antisocial acts) [https://www.youtube.com/embed/fQlO1eFA_r8?rel=0&showinfo=0]

*In the weeks that followed, several things happened to the young man. He came home to find that the dog, tired from chasing squirrels, was asleep on the rug. On payday, while he was helping the street vendor move her cart, his wallet fell out of his pocket. He noticed, picked up his wallet, and put it in a deeper pocket. The old woman who lived next door left her door ajar as she listened to music on the radio in the morning. Lastly, the homeless girl sat next to her mother and did her homework in the afternoon.*

6. Elevation Scale (see Study 1)

7. Study Checks

   Items:

- Sound problems
- Playback problems
- Attention self-report (Did you watch all the videos / Did you pay attention to the passage at the end of the second video?)
- Video/passage attention check (Which of the following did you see in the second video?)
- General attention check (If you go outside on a clear day and look up, what color should the sky usually be?)

8. Demographics 2 (see Study 1)

**Study 3**

1. Idealism Scale (Sparks et al., n.d.)

Instructions: Please think about the kinds of people who you tend to encounter in your daily life, but don?t know very well. These people are not family or friends or someone you've had many conversations with. They are acquaintances or strangers who seem typical of the social circle(s) that you are a part of. We will call these people your "broader community" – please think about this group when you answer these questions.

Please rate your level of agreement with the following statements about your broader community. 1-strongly disagree to 7-strongly agree

Items:

- Life is beautiful in my broader community.
- In my broader community, it is important to make sure you are not exploited.
- If there's something I need that my family and close friends cannot help with, I know that my broader community will help.
- I prefer to keep a distance from most people in my broader community.
- I am treated right by my broader community.
- My broader community is full of great people.
- In my broader community, people do not trust each other.
- In my broader community, people do not try to take advantage of you, even if they get the chance.
- Most people are basically honest in my broader community.
- I am pessimistic about my broader community.

2. Presentation of an affectively neutral video (see Study 1)

3. Elevation Scale (see Study 1)

4. Demographics 1 (see Study 1)

5. Presentation of vignette stimuli (participants in all conditions read the same base vignette, and then are randomly assigned to one of six possible ending conditions)

Base vignette:

*Jim is in his early twenties, and lives in large city where he has many friends and acquaintances. One day, Jim walks down the street on his way to work. City life bustles around him; street vendors hawk breakfast foods, the occasional dog passes by, and parents walk their children to school. Jim takes this route every day, and he's familiar with the sights, people, and sounds of his neighborhood. He notices an older street vendor struggling to push his food cart onto the curb; he approaches and, after a quiet nod, helps him get the heavy cart up onto the sidewalk before continuing on his way. He stops at his favorite cafe for a quick bite to eat before work, and sits outside on the patio. While he is eating and chatting with a friend on Facebook, a worryingly thin stray dog cautiously approaches, begging for food. Although Jim works hard, his job doesn't pay very well, and his morning cafe stops are one of the few small luxuries he affords himself. He looks down at the hungry stray, gazes for a moment at his breakfast, then divides the meal in half and shares it with the dog.*

*The next morning, Jim is on the phone with his cousin who lives across town when he realizes that he doesn't hear the usual noises from the apartment next door. An elderly man, who Jim has never met, moved in next door a month ago, and normally he turns on the TV in the morning. Concerned, he goes over to his apartment to check on him. He discovers that the old man is okay, but Jim still worries that he's having difficulty taking care of himself. That evening, after work, Jim stops at the store, buys several bags of groceries, and leaves them outside his neighbor's door. The next day, the elderly man tries to pay him back, but Jim refuses, knowing that his neighbor has little money of his own; he accepts a hug instead.*

*Jim continues these acts of kindness throughout his neighborhood. For example, the bus he rides to work is usually crowded, and Jim always gives up his seat for older people who are standing, or for other passengers who look tired or sick.*

*One day, while walking to work, Jim notices a mother and her little girl begging on a street corner, dressed in dirty clothes with holes, and sitting on a ragged piece of cardboard. Other people walk past, ignoring them. Jim pauses, then opens his wallet and pulls out the largest bills has, putting them in the cup held by the young daughter. The little girl looks sad and ashamed, and takes Jim's money without making eye contact. Over the weeks that follow, Jim comes back to give the mother and child cash whenever he can. One day, on his way to work, when Jim approaches their usual spot on the corner, he sees that the woman is alone. Deeply worried, he starts looking around for the little girl, and rushes up to the mother to make sure everything is okay. Then, as he gets closer, he sees the daughter walking down the sidewalk toward her mother, dressed in brand new school clothes and carrying a backpack. She smiles proudly when she sees Jim. He realizes that the mother had been able to use his donations to help her daughter in school. Jim looks at the woman and she smiles, her eyes filling with tears as she looks at the kind man who has helped her family without asking for anything in return.*

Vignette Endings:

- Pay-It-Back condition (direct reciprocation by the beneficiaries)

*In the weeks that followed, several things happened to Jim. While cooking dinner, he sat down to rest, and, tired from a long day, fell asleep. He was awakened by the stray dog barking and pawing at him, and realized that the food on the stove had caught fire. Jim was able to put out the fire just in time.*

*The next week, Jim threw out his back helping the street vendor with his cart. Jim visited the local clinic, where they told him to rest and recover. The street vendor brought him*

*meals every evening, and gave him pain medication and hot compresses, and Jim soon recovered. Soon after, the elderly man who lived next door observed that Jim accidentally left his door ajar when leaving for work, so the neighbor locked the door and closed it for him. Lastly, on payday, Jim's wallet fell out of his pocket as he walked home from work. The homeless girl, sitting with her mother across the street, saw what happened. The girl ran over, picked up the wallet, caught up with Jim, and returned it to him with a smile.*

- Pay-It-Forward condition (prosocial actions directed at third parties by the beneficiaries)

*In the weeks that followed, several things happened in Jim's neighborhood. While prepping food, the cook from Jim's favorite cafe sat down to rest, and, tired from a long day, fell asleep. He was awakened by the stray dog barking and pawing at him, and realized that the food on the stove had caught fire. The cook was able to put out the fire just in time.*

*The next week, a local shopkeeper strained his back moving some heavy boxes. The shopkeeper visited the local clinic, where they told him to rest and recover. The street vendor, who usually set up his cart in front of the shopkeeper's store, brought him food every evening, and arranged for his sister to give him medication and hot compresses; he soon recovered. Also, Jim's elderly neighbor observed that another resident of the building accidentally left her door ajar when leaving for work, so he locked the door and closed it for the neighbor. Lastly, on payday, after he paid for his lunch at the cafe, a stranger accidentally forgot his wallet. The homeless girl, sitting with her mother across the street, saw what happened. The girl ran over, picked up the wallet, caught up with the stranger, and returned it to the stranger with a smile.*

- Lone-Altruist condition (additional prosocial acts by the protagonist)

*In the weeks that followed, several things happened to Jim. While prepping food, the cook from Jim's favorite cafe sat down to rest, and, tired from a long day, fell asleep. While*

*the cook was asleep, his stove caught fire. Jim, enjoying an after-work meal with some friends, saw the smoke and put out the fire just in time.*

*The next week, the street vendor threw out his back moving heavy boxes of produce. The vendor visited the local clinic, where they told him to rest and recover. Jim brought him meals every evening, and gave him pain medication and hot compresses, and the street vendor soon recovered. Soon after, Jim observed that his elderly neighbor accidentally left his door ajar when leaving for work. Worried, Jim locked the door and closed it for him. Lastly, a stranger was eating lunch at the cafe on payday; after the stranger paid for her lunch, she accidentally forgot her wallet. Jim saw what happened, picked up the wallet, and caught up with the stranger to return her wallet.*

- Exploited condition (protagonist exploited by his beneficiaries)

*In the weeks that followed, several things happened to Jim. The stray dog ran up to his table at the cafe one morning and grabbed Jim's entire breakfast before sprinting off. Then, while he was helping the street vendor move his cart, Jim twisted his ankle painfully. The street vendor just kept pushing the cart down the road as Jim limped behind him, alone.*

*The next week, the old man who lived next door observed that Jim accidentally left his door ajar when leaving for work. While Jim was away, the old man snuck inside and stole Jim's small television. Lastly, on payday, while Jim was walking down the street, his wallet fell out of his pocket. The young homeless girl saw what happened, and, when Jim wasn't looking, she quickly picked up his wallet, slipped the cash in her pocket, and dropped the wallet back on the street.*

- Martyr condition (protagonist continues acting prosocially following exploitation by his beneficiaries)

*In the weeks that followed, several things happened to Jim. The stray dog ran up to his*

*table at the cafe one morning and grabbed Jim's entire breakfast before sprinting off. Then, while he was helping the street vendor move his cart, Jim twisted his ankle painfully. The street vendor just kept pushing the cart down the road as Jim limped behind him, alone. Later, the street vendor threw out his back moving heavy boxes of produce. The vendor visited the local clinic, where they told him to rest and recover. Jim brought him meals every evening, and gave him pain medication and hot compresses, and the street vendor recovered.*

*The next week, the old man who lived next door observed that Jim accidentally left his door ajar when leaving for work. The neighbor snuck inside, but Jim returned home and caught him stealing his small television. Soon after, Jim observed that his elderly neighbor accidentally left his door ajar when leaving for work. Worried, Jim locked the door and closed it for him.*

*Lastly, on payday, while Jim was walking down the street, his wallet fell out of his pocket. The young homeless girl saw what happened but said nothing, and, when she thought Jim wasn't looking, she quickly picked up his wallet, slipped the cash in her pocket, and dropped the wallet back on the street. Jim saw what she did but picked up the wallet and said nothing. when Jim saw the young homeless girl and her mother begging on the street, he put money in their cup.*

- No-Additional-Information condition (no further prosocial or antisocial acts)

*In the weeks that followed, several things happened to Jim. Eating at his local cafe, he chatted with the chef about soccer. Walking home, Jim noticed a group of dogs playing in the local park. On payday, his wallet fell out of his pocket. Jim noticed, picked up his wallet, and put it in a deeper pocket. Finally, while moving some furniture around in his apartment, Jim threw out his back. Jim visited the local clinic, where they told him to rest and recover. After a few weeks of taking it easy, Jim felt back to normal.*

6. Elevation Scale (see Study 1)

7. Study Checks

   Items:

- Attention self-report (Did you pay attention to the passage?)
- Vignette attention check (Which of the following did you read in the passage?)
- General attention check (If you go outside on a clear day and look up, what color should the sky usually be?)

8. Demographics 2 (see Study 1)

Table 10

*Scale reliabilities (alphas) by study.*

| | 1 | 2 | 3 |
|---|---|---|---|
| idealism | 0.93 | 0.93 | 0.82 |
| elevation | 0.97 | 0.96 | 0.95 |
| elevation_pre | 0.95 | 0.94 | 0.98 |
| somatic | 0.88 | 0.88 | 0.85 |
| somatic_pre | 0.81 | 0.80 | 0.94 |
| folk affect | 0.96 | 0.96 | 0.94 |
| folk affect pre | 0.93 | 0.93 | 0.96 |
| prosocial_motives | 0.95 | 0.93 | 0.91 |
| prosocial_motives_pre | 0.86 | 0.85 | 0.93 |
| positive_affect | 0.86 | 0.83 | 0.83 |
| positive_affect_pre | 0.85 | 0.83 | 0.92 |

*Note.* Emotion measured before the experimental manipulation labelled pre.

## Scale Reliabilities

Internal reliability information for the scale measures used in these studies is reported in Table 10

## Power Analysis and Sample Size

Study 2 uses modified versions of the prosocial story. In study 1, the full Unsung Hero video elicited greater elevation levels than other prosocial conditions; the associated effect size ($d$ = -0.51) can be used in a post-hoc power estimation for contrasts of emotion levels between conditions of Study 2 that added anti-social information verses those that did not. Based on the final sample of the latter study, power to detect an effect of this size, at $p$ = .01, was nearly 100%.

## Elimination decisions

Tables 11 to 20 summarize raw response frequencies among all participants for various variables used to filter down to our final samples. After data were filtered based on those criteria, a final filter was applied based on suspiciously quick finishing (study 1 = 86; study 2 = 4 ; study 3 = 8; see R code for details).

Study 3 included an attention check question ("check2") that many participants failed. In retrospect it was a bad question because it asked the participant to recall a minor details ("In the passage you read, who does Jim talk on the phone with?"). Jim was on the phone with his cousin, but the specific person he spoke to was not relevant to anything else in the story. (The story included this detail to convey that Jim is not a lonely or isolated person.) All of the answer options are various types of close partners or family members, relationship types that might become conflated with "cousin" for such a minor character. So, we did not filter based on this variable.

Table 11

*Response frequencies for filtering variables - Study 1*

| | device | audio1 | audio2 | playback | check |
|---|---|---|---|---|---|
| | Desktop computer: 623 | no : 32 | no : 14 | 1 :1749 | blue:1800 |
| | Laptop computer :1069 | Other - Write In: 11 | yes :1199 | 2 : 42 | pink: 3 |
| | Phone : 49 | yes :1758 | NA's: 591 | 3 : 6 | NA's: 1 |
| | Tablet : 60 | NA's : 3 | – | NA's: 7 | – |
| | NA's : 3 | – | – | – | – |

Table 12

*Response frequencies for filtering variables applying to all conditions - Study 2 (1 of 2)*

| | device | audio1 | audio2 |
|---|---|---|---|
| | Desktop computer:258 | no : 12 | no : 8 |
| | Laptop computer :330 | Other - Write In: 10 | Other - Write In: 2 |
| | Phone : 11 | yes :581 | yes :591 |
| | Tablet : 7 | NA's : 4 | NA's : 6 |
| | NA's : 1 | – | – |
| | – | – | – |
| | – | – | – |

Table 13

*Response frequencies for filtering variables applying to all conditions - Study 2 (2 of 2)*

| | playback | check_blue_4 | bananas_1_check |
|---|---|---|---|
| | 1 :562 | antelope: 1 | a cat : 15 |
| | 2 : 34 | blue :604 | a school: 17 |
| | 3 : 8 | pink : 1 | bananas :550 |
| | NA's: 3 | snack : 1 | boats : 6 |
| | – | – | cubicles: 5 |
| | – | – | mango : 11 |
| | – | – | NA's : 3 |

Table 14

*Response frequencies for filtering variable specific to condition 'Lone Altruist' - Study 2*

| | wallet_4 |
|---|---|
| | his checkbook: 2 |
| | his jacket : 1 |
| | his phone : 1 |
| | his wallet :121 |

Table 15

*Response frequencies for filtering variable specific to condition 'Exploitation' - Study 2*

| | girl1 |
|---|---|
| | begging on the street :29 |
| | eating an ice cream cone : 2 |
| | going to school : 5 |
| | playing with neighborhood kids: 2 |
| | stealing wallets :60 |

Table 16

*Response frequencies for filtering variable specific to condition 'Pay-it-Back' - Study 2*

| | wallet_2 |
|---|---|
| | his jacket: 2 |
| | his keys : 2 |
| | his wallet:83 |

Table 17

*Response frequencies for filtering variable specific to condition 'Pay-it-Forward' - Study 2*

| | wallet_3 |
|---|---|
| | his checkbook : 1 |
| | his credit card: 1 |
| | his jacket : 3 |
| | his wallet :104 |

Table 18

*Response frequencies for filtering variable specific to condition 'No-Additional-Info' - Study 2*

| | girl2 |
|---|---|
| | begging on the street :14 |
| | doing homework :62 |
| | going to school : 5 |
| | playing with neighborhood kids: 1 |
| | stealing wallets : 1 |

Table 19

*Response frequencies for filtering variable specific to condition 'Martyr' - Study 2*

| | girl3 |
|---|---|
| | begging on the street:62 |
| | doing homework : 2 |
| | going to school : 4 |
| | stealing wallets :37 |

Table 20

*Response frequencies for filtering variables - Study 3*

| | device | check1 | check2 | check3 |
|---|---|---|---|---|
| | Desktop computer:273 | a cat : 11 | his best friend: 92 | no : 9 |
| | Laptop computer :304 | a school: 13 | his brother : 67 | yes :561 |
| | Phone : 17 | a train :556 | his cousin :308 | yes, but I did not pay close attention: [illegible] |
| | Tablet : 9 | boats : 7 | his girlfriend : 22 | NA's : 3 |
| | NA's : 1 | cubicles: 12 | his grandmother: 26 | – |
| | – | mangoes : 2 | his mother : 83 | – |
| | – | NA's : 3 | NA's : 6 | – |
